# Supplementary material for: Multi‐dataset identification of innovative feature genes and molecular mechanisms in keratoconus
Source: J Cell Mol Med. 2024 Sep 19;28(18):e70079. doi: 10.1111/jcmm.70079 (PMC11412914; doi:10.1111/jcmm.70079)
Supplement: Supplementary file 2 — Material S2. [file JCMM-28-e70079-s002.zip › 7.GSEA analysis/5.RNF217-AS1/KEGG_CHEMOKINE_SIGNALING_PATHWAY.html]

Details for gene set KEGG\_CHEMOKINE\_SIGNALING\_PATHWAY[GSEA]

|  || Dataset | RNF217-AS1.group.cls#h\_versus\_l |
| Phenotype | group.cls#h\_versus\_l |
| Upregulated in class | l |
| GeneSet | KEGG\_CHEMOKINE\_SIGNALING\_PATHWAY |
| Enrichment Score (ES) | -0.42003277 |
| Normalized Enrichment Score (NES) | -1.4785541 |
| Nominal p-value | 0.03373016 |
| FDR q-value | 0.31568635 |
| FWER p-Value | 0.79 |
Table: GSEA Results Summary

  

Fig 1: Enrichment plot: KEGG\_CHEMOKINE\_SIGNALING\_PATHWAY      
 Profile of the Running ES Score & Positions of GeneSet Members on the Rank Ordered List

  

| SYMBOL | TITLE | RANK IN GENE LIST | RANK METRIC SCORE | RUNNING ES | CORE ENRICHMENT || 1 | ITK | na | 416 | 0.552 | 0.0079 | No |
| 2 | PF4 | na | 652 | 0.498 | 0.0191 | No |
| 3 | PIK3R5 | na | 1058 | 0.434 | 0.0231 | No |
| 4 | HCK | na | 2257 | 0.330 | 0.0004 | No |
| 5 | CCR10 | na | 2315 | 0.327 | 0.0105 | No |
| 6 | ADCY2 | na | 2970 | 0.293 | 0.0022 | No |
| 7 | CCR6 | na | 3752 | 0.261 | -0.0110 | No |
| 8 | BCAR1 | na | 4327 | 0.240 | -0.0189 | No |
| 9 | IKBKB | na | 4368 | 0.238 | -0.0114 | No |
| 10 | ADCY4 | na | 4384 | 0.238 | -0.0033 | No |
| 11 | CCL14 | na | 4571 | 0.231 | -0.0003 | No |
| 12 | CCL22 | na | 4683 | 0.228 | 0.0048 | No |
| 13 | ADCY5 | na | 4777 | 0.225 | 0.0102 | No |
| 14 | XCL1 | na | 5292 | 0.206 | 0.0028 | No |
| 15 | BRAF | na | 5517 | 0.199 | 0.0035 | No |
| 16 | ADCY7 | na | 5579 | 0.197 | 0.0088 | No |
| 17 | CXCR2 | na | 5883 | 0.187 | 0.0068 | No |
| 18 | CCL20 | na | 6274 | 0.175 | 0.0019 | No |
| 19 | PIK3CD | na | 6778 | 0.159 | -0.0069 | No |
| 20 | CCL19 | na | 6982 | 0.153 | -0.0073 | No |
| 21 | PLCB3 | na | 7444 | 0.141 | -0.0155 | No |
| 22 | GNAI3 | na | 7550 | 0.139 | -0.0135 | No |
| 23 | CCR3 | na | 8121 | 0.125 | -0.0255 | No |
| 24 | NCF1 | na | 8249 | 0.122 | -0.0248 | No |
| 25 | CCR9 | na | 8540 | 0.115 | -0.0290 | No |
| 26 | RASGRP2 | na | 8587 | 0.114 | -0.0262 | No |
| 27 | VAV2 | na | 9125 | 0.103 | -0.0380 | No |
| 28 | CCR4 | na | 9412 | 0.097 | -0.0428 | No |
| 29 | SOS1 | na | 9467 | 0.096 | -0.0409 | No |
| 30 | CCL15 | na | 10100 | 0.085 | -0.0561 | No |
| 31 | CXCL8 | na | 10297 | 0.081 | -0.0589 | No |
| 32 | ROCK1 | na | 10313 | 0.081 | -0.0564 | No |
| 33 | GNG8 | na | 10331 | 0.081 | -0.0539 | No |
| 34 | CCL23 | na | 10573 | 0.077 | -0.0581 | No |
| 35 | CCL11 | na | 10610 | 0.076 | -0.0564 | No |
| 36 | ADCY1 | na | 10624 | 0.076 | -0.0540 | No |
| 37 | PTK2 | na | 10929 | 0.071 | -0.0603 | No |
| 38 | GSK3B | na | 10962 | 0.071 | -0.0586 | No |
| 39 | PRKCZ | na | 11072 | 0.069 | -0.0593 | No |
| 40 | PARD3 | na | 11290 | 0.066 | -0.0632 | No |
| 41 | PIK3CB | na | 11439 | 0.064 | -0.0652 | No |
| 42 | KRAS | na | 11445 | 0.064 | -0.0630 | No |
| 43 | ARRB2 | na | 11624 | 0.061 | -0.0660 | No |
| 44 | XCR1 | na | 11829 | 0.058 | -0.0698 | No |
| 45 | CXCR5 | na | 12083 | 0.054 | -0.0751 | No |
| 46 | CCL26 | na | 12447 | 0.049 | -0.0839 | No |
| 47 | CXCL16 | na | 12558 | 0.048 | -0.0853 | No |
| 48 | RAC2 | na | 12945 | 0.043 | -0.0949 | No |
| 49 | MAPK1 | na | 13362 | 0.038 | -0.1056 | No |
| 50 | PIK3CA | na | 14021 | 0.031 | -0.1236 | No |
| 51 | PTK2B | na | 14053 | 0.030 | -0.1234 | No |
| 52 | MAPK3 | na | 14127 | 0.029 | -0.1244 | No |
| 53 | CXCL12 | na | 14162 | 0.029 | -0.1244 | No |
| 54 | CXCL3 | na | 14767 | 0.022 | -0.1411 | No |
| 55 | WAS | na | 14820 | 0.021 | -0.1418 | No |
| 56 | CCL16 | na | 14923 | 0.020 | -0.1440 | No |
| 57 | RHOA | na | 14935 | 0.020 | -0.1436 | No |
| 58 | GNAI2 | na | 14991 | 0.019 | -0.1445 | No |
| 59 | CCL17 | na | 15056 | 0.018 | -0.1457 | No |
| 60 | GNG13 | na | 15457 | 0.014 | -0.1568 | No |
| 61 | PRKCD | na | 15568 | 0.013 | -0.1595 | No |
| 62 | RAP1A | na | 15733 | 0.011 | -0.1638 | No |
| 63 | AKT2 | na | 15816 | 0.011 | -0.1658 | No |
| 64 | PXN | na | 15819 | 0.011 | -0.1655 | No |
| 65 | HRAS | na | 15878 | 0.010 | -0.1668 | No |
| 66 | CCL24 | na | 16003 | 0.009 | -0.1701 | No |
| 67 | PIK3R1 | na | 16145 | 0.007 | -0.1739 | No |
| 68 | RAP1B | na | 16191 | 0.007 | -0.1750 | No |
| 69 | CCL18 | na | 16419 | 0.004 | -0.1814 | No |
| 70 | TIAM1 | na | 16433 | 0.004 | -0.1816 | No |
| 71 | JAK3 | na | 16463 | 0.004 | -0.1823 | No |
| 72 | CHUK | na | 16613 | 0.003 | -0.1865 | No |
| 73 | GNB2 | na | 16830 | 0.000 | -0.1927 | No |
| 74 | GNG7 | na | 17038 | -0.002 | -0.1987 | No |
| 75 | ADCY9 | na | 17206 | -0.004 | -0.2034 | No |
| 76 | NRAS | na | 17304 | -0.005 | -0.2060 | No |
| 77 | CCL3L3 | na | 17746 | -0.008 | -0.2185 | No |
| 78 | WASL | na | 17958 | -0.010 | -0.2242 | No |
| 79 | CDC42 | na | 18054 | -0.011 | -0.2265 | No |
| 80 | CSK | na | 18243 | -0.013 | -0.2315 | No |
| 81 | AKT1 | na | 18280 | -0.013 | -0.2321 | No |
| 82 | RAC1 | na | 18357 | -0.014 | -0.2338 | No |
| 83 | NFKBIB | na | 18425 | -0.015 | -0.2352 | No |
| 84 | ROCK2 | na | 18737 | -0.018 | -0.2435 | No |
| 85 | SHC1 | na | 18911 | -0.019 | -0.2478 | No |
| 86 | RAF1 | na | 18969 | -0.020 | -0.2488 | No |
| 87 | GSK3A | na | 18988 | -0.020 | -0.2486 | No |
| 88 | CCR8 | na | 19202 | -0.022 | -0.2539 | No |
| 89 | ADCY6 | na | 19355 | -0.024 | -0.2575 | No |
| 90 | FOXO3 | na | 19500 | -0.025 | -0.2607 | No |
| 91 | GNG5 | na | 19682 | -0.027 | -0.2650 | No |
| 92 | SOS2 | na | 19710 | -0.027 | -0.2648 | No |
| 93 | CCL8 | na | 19827 | -0.028 | -0.2671 | No |
| 94 | GRK5 | na | 19852 | -0.028 | -0.2668 | No |
| 95 | PF4V1 | na | 20148 | -0.031 | -0.2742 | No |
| 96 | CCL27 | na | 20261 | -0.033 | -0.2763 | No |
| 97 | GNG12 | na | 20494 | -0.035 | -0.2817 | No |
| 98 | NFKB1 | na | 20531 | -0.035 | -0.2815 | No |
| 99 | CXCL2 | na | 20623 | -0.036 | -0.2828 | No |
| 100 | IKBKG | na | 20686 | -0.037 | -0.2833 | No |
| 101 | CRK | na | 21000 | -0.040 | -0.2909 | No |
| 102 | CXCL14 | na | 21176 | -0.042 | -0.2944 | No |
| 103 | PLCB4 | na | 21374 | -0.044 | -0.2986 | No |
| 104 | STAT1 | na | 21406 | -0.044 | -0.2979 | No |
| 105 | SHC4 | na | 21413 | -0.044 | -0.2964 | No |
| 106 | GNG10 | na | 21442 | -0.045 | -0.2956 | No |
| 107 | RELA | na | 21550 | -0.046 | -0.2971 | No |
| 108 | ADCY8 | na | 21903 | -0.050 | -0.3055 | No |
| 109 | GNB1 | na | 22143 | -0.052 | -0.3105 | No |
| 110 | CXCL5 | na | 22147 | -0.052 | -0.3087 | No |
| 111 | CCL4L2 | na | 22264 | -0.054 | -0.3101 | No |
| 112 | CXCR4 | na | 22323 | -0.054 | -0.3098 | No |
| 113 | GRB2 | na | 22374 | -0.055 | -0.3093 | No |
| 114 | STAT2 | na | 22528 | -0.056 | -0.3117 | No |
| 115 | STAT3 | na | 22707 | -0.058 | -0.3147 | No |
| 116 | ADCY3 | na | 22794 | -0.059 | -0.3151 | No |
| 117 | PIK3R2 | na | 22894 | -0.061 | -0.3157 | No |
| 118 | PRKACA | na | 23066 | -0.063 | -0.3184 | No |
| 119 | STAT5B | na | 23102 | -0.063 | -0.3172 | No |
| 120 | MAP2K1 | na | 23215 | -0.064 | -0.3181 | No |
| 121 | GNG3 | na | 23242 | -0.065 | -0.3165 | No |
| 122 | GRK6 | na | 23357 | -0.066 | -0.3174 | No |
| 123 | PAK1 | na | 23429 | -0.067 | -0.3170 | No |
| 124 | GNG2 | na | 23513 | -0.068 | -0.3169 | No |
| 125 | CRKL | na | 23841 | -0.073 | -0.3238 | No |
| 126 | NFKBIA | na | 24662 | -0.085 | -0.3444 | No |
| 127 | VAV3 | na | 24686 | -0.086 | -0.3420 | No |
| 128 | LYN | na | 24892 | -0.089 | -0.3447 | No |
| 129 | GNG4 | na | 26233 | -0.111 | -0.3795 | No |
| 130 | CXCL13 | na | 26277 | -0.112 | -0.3767 | No |
| 131 | PRKACB | na | 26457 | -0.116 | -0.3777 | No |
| 132 | CCR2 | na | 26949 | -0.125 | -0.3874 | No |
| 133 | JAK2 | na | 27229 | -0.131 | -0.3907 | No |
| 134 | CCL28 | na | 27483 | -0.136 | -0.3931 | No |
| 135 | GRK1 | na | 27644 | -0.140 | -0.3927 | No |
| 136 | TIAM2 | na | 27714 | -0.142 | -0.3896 | No |
| 137 | PPBP | na | 27821 | -0.144 | -0.3875 | No |
| 138 | CCR7 | na | 28256 | -0.155 | -0.3944 | No |
| 139 | GNAI1 | na | 28406 | -0.159 | -0.3930 | No |
| 140 | CXCL11 | na | 29011 | -0.176 | -0.4041 | No |
| 141 | GRK4 | na | 29343 | -0.186 | -0.4069 | No |
| 142 | CCL2 | na | 29797 | -0.200 | -0.4128 | Yes |
| 143 | GNB5 | na | 29969 | -0.205 | -0.4103 | Yes |
| 144 | SHC3 | na | 30112 | -0.210 | -0.4068 | Yes |
| 145 | GNGT2 | na | 30229 | -0.214 | -0.4025 | Yes |
| 146 | PIK3CG | na | 30335 | -0.218 | -0.3976 | Yes |
| 147 | CXCL6 | na | 30464 | -0.222 | -0.3933 | Yes |
| 148 | ARRB1 | na | 30690 | -0.230 | -0.3915 | Yes |
| 149 | CCR5 | na | 30766 | -0.233 | -0.3852 | Yes |
| 150 | CCL21 | na | 30779 | -0.233 | -0.3771 | Yes |
| 151 | CCL3 | na | 30858 | -0.236 | -0.3709 | Yes |
| 152 | PLCB2 | na | 31149 | -0.248 | -0.3703 | Yes |
| 153 | CXCR6 | na | 31340 | -0.256 | -0.3665 | Yes |
| 154 | PRKX | na | 31362 | -0.257 | -0.3578 | Yes |
| 155 | ELMO1 | na | 31391 | -0.258 | -0.3493 | Yes |
| 156 | CCL7 | na | 31544 | -0.266 | -0.3441 | Yes |
| 157 | GNB3 | na | 31630 | -0.270 | -0.3368 | Yes |
| 158 | CXCR1 | na | 31763 | -0.277 | -0.3306 | Yes |
| 159 | AKT3 | na | 31832 | -0.281 | -0.3224 | Yes |
| 160 | SHC2 | na | 31857 | -0.283 | -0.3128 | Yes |
| 161 | CX3CL1 | na | 32369 | -0.315 | -0.3162 | Yes |
| 162 | PLCB1 | na | 32371 | -0.315 | -0.3049 | Yes |
| 163 | FGR | na | 32401 | -0.317 | -0.2942 | Yes |
| 164 | CXCR3 | na | 32796 | -0.345 | -0.2932 | Yes |
| 165 | CCL5 | na | 32851 | -0.349 | -0.2821 | Yes |
| 166 | PIK3R3 | na | 32895 | -0.352 | -0.2706 | Yes |
| 167 | PRKACG | na | 33174 | -0.374 | -0.2651 | Yes |
| 168 | CXCL1 | na | 33320 | -0.387 | -0.2553 | Yes |
| 169 | CCL13 | na | 33447 | -0.401 | -0.2445 | Yes |
| 170 | CCR1 | na | 33677 | -0.429 | -0.2356 | Yes |
| 171 | CCL1 | na | 33777 | -0.443 | -0.2224 | Yes |
| 172 | PREX1 | na | 33921 | -0.466 | -0.2097 | Yes |
| 173 | CX3CR1 | na | 34012 | -0.484 | -0.1947 | Yes |
| 174 | CXCL10 | na | 34045 | -0.491 | -0.1779 | Yes |
| 175 | GNGT1 | na | 34058 | -0.495 | -0.1604 | Yes |
| 176 | VAV1 | na | 34083 | -0.499 | -0.1430 | Yes |
| 177 | DOCK2 | na | 34118 | -0.506 | -0.1257 | Yes |
| 178 | PRKCB | na | 34175 | -0.520 | -0.1085 | Yes |
| 179 | CCL25 | na | 34245 | -0.539 | -0.0910 | Yes |
| 180 | GRK7 | na | 34385 | -0.592 | -0.0736 | Yes |
| 181 | GNG11 | na | 34508 | -0.660 | -0.0532 | Yes |
| 182 | GNB4 | na | 34615 | -0.750 | -0.0292 | Yes |
| 183 | CXCL9 | na | 34683 | -0.896 | 0.0013 | Yes |
Table: GSEA details [plain text format]

  

Fig 2: KEGG\_CHEMOKINE\_SIGNALING\_PATHWAY      
 Blue-Pink O' Gram in the Space of the Analyzed GeneSet

  

Fig 3: KEGG\_CHEMOKINE\_SIGNALING\_PATHWAY: Random ES distribution      
 Gene set null distribution of ES for **KEGG\_CHEMOKINE\_SIGNALING\_PATHWAY**
